# Circadian Abnormalities in Motor Activity in a BAC Transgenic Mouse Model of Huntington’s Disease

**DOI:** 10.1371/currents.RRN1225

**Published:** 2011-04-05

**Authors:** Stephen Oakeshott, Fuat Balci, Igor Filippov, Carol Murphy, Russell Port, David Connor, Ahmad Paintdakhi, Joseph LeSauter, Liliana Menalled, Sylvie Ramboz, Seung Kwak, David Howland, Rae Silver, Dani Brunner

**Affiliations:** ^*^PsychoGenics, Inc.; ^†^Princeton University; ^††^Barnard College; ^¶¶^CHDI Foundation; ^***^Barnard College and Columbia University and ^†††^PsychoGenics/Columbia Univ.

## Abstract

Huntington’s disease (HD) is a progressive neurodegenerative disease marked by psychiatric and motor problems. Recently, these findings have been extended to deficits in sleep and circadian function that can be observed in HD patients and in HD mouse models, with abnormal sleep patterns correlating with symptom severity in patients. Here, we studied the behavior of the BAC HD mouse model using an 24/7 automated system; the results indicate significant lengthening of the circadian period in the mutant mice. These results reinforce previous findings in HD models and symptomatic HD patients, indicating that circadian dysfunction is a core feature of HD.

## **Introduction**

In the last few years, deficits in sleep and circadian function have been observed in HD patients  and in HD mouse models [1] [2] [3].  As expected, deficits in sleep-wake patterns have profound consequences in many physiological functions. In HD patients, abnormal sleep patterns correlate with symptom severity, specifically in depression and cognitive dysfunction, and in caudate atrophy [4] [5] [6]. Here, we studied the behavior of the BAC HD model [7] [8] using an 24/7 automated home-cage system and custom-built computer vision assessment of behavior and in a visual discrimination task. The results indicate a significant lengthening of the circadian period in the mutants without any visual impairment. The results reinforce previous findings in the R6/2 mouse and in a transgenic rat model of HD [2] [3], indicating that circadian dysfunction is a fundamental feature of HD.

Huntington’s disease (HD) is a progressive neurodegenerative disease caused by a CAG expansion of the huntingtin gene. It is characterized by progressive dysfunction of fronto-striatal circuits, hypothalamus, and loss of the striatal medium spiny neurons. While HD is marked mainly by psychiatric and motor problems [9], it is also clear that metabolic and endocrine deficits accompany overt symptoms [10].

HD patients have abnormal sleep patterns, with increased sleep onset latency, frequent awakening, and changes in the normal EEG pattern. These responses correlate with symptom severity and caudate atrophy [5] [6]. During sleep, HD patients show more motor activity than controls [11], while changes in melatonin secretion are correlated with sleep  disturbances [12]. Hypothalamic pathology results in abnormalglucocorticoid feedback and HPA axis hyperactivity [13] [14]. Despite significant changes in circadian function, some studies have failed to find circadian changes in endocrine function.For example, normal circadian rhythms of prolactin secretion were reported for a small group of HD patients[15].

The R6/2 HD mouse recapitulates the disrupted day-night activity pattern seen in HD patients[3]. In this mouse model, the disintegration of circadian rhythms in behavioral readouts was accompanied by abnormal expression of the clock genes mPer2 and mBmal1, and mBmal1 transcriptional target prokineticin 2 in the suprachiasmatic nuclei (SCN) [3] [16]. In the motor cortex and striatum, circadian gene expression patterns were also abnormal. Circadian rhythms in peripheral metabolic pathways are also affected in R6/2 mice, possibly reflecting circadian desynchronization secondary to SCN circadian dysfunction and reduced entrainment due to abnormal feeding patterns [16].

Here, we studied the diurnal and circadian rhythms and visual discrimination responses of a different model, the BAC HD mouse, which has been shown to recapitulate some of the HD pathophysiology and behavioral abnormalities [7] [8].  Although the R6/2 model is the best-characterized and most widely-used model of HD [17] [18] [19] [20] [21], research has actively focused in alternative models as the R6/2 expresses only the short N-terminal mutant fragment of the huntingtin protein. The BAC HD transgenic model, in contrast, expresses the full-length mutant human huntingtin gene under the control of the endogenous huntingtin promoter inserted in a bacterial artificial chromosome [7]. This model carries 97 to 98 CAA-CAG repeats encoding a pure polyQ stretch within the human huntingtin protein.  This mixed repeat sequence has been shown to provide stable repeat length across generations [7]. These BAC103 mice display progressive and robust motor deficits with cortical and striatal atrophy around 1 year of age. 

We used an automated home-cage system that allowed continuous computer vision assessment of behavior for virtually 6 full days to detect possible circadian abnormalities. In addition, we assessed mice in a visual discrimination task, the cued two-choice swim tank, to evaluate the possibility of major visual dysfunction in the mutants, a phenotype that could indirectly affect circadian function.

## **Materials and Methods**

***Subjects*** *.* Two cohorts of 64 female mice, bred at the Jackson Laboratory, served as the primary subjects in this study. Mutant mice carried the full-length human mutant huntingtin, with 97 glutamine repeats under the control of endogenous httregulatory machinery on a bacterial artificial chromosome (BAC). All mice tested here were generated on an FVB/n x C57Bl6 F1, created by crossing BAC hemizygous  FVB/n male animals with wildtype C57Bl6 female animals. The congenic BAC FVB/n line used for these breedings, originally sourced from the laboratory of X. William Yang at the UCLA David Geffen School of Medicine, Los Angeles, is maintained by crossing hemizygous male BAC mice with FVB/n female animals. Each cohort was comprised of 32 BAC mice and 32 WT controls. Mice were implanted with RFID electronic chips (DataMars, OH) for identification. The animals were split into homogenous groups of eight by genotype and housed in OptiRAT® cages (Animal Care Systems, CO) for several months prior to the experiment, with free access to water and to standard 5001 lab chow except as described. A third cohort of 67 week old female mice, 20 BAC and 19 WT controls, were used as subjects for the two choice swim tank study. These animals were housed in smaller optiMICE® (Animal Care Systems, CO) cages in mixed genotype groups of 4 to 5 mice. The colony was maintained on a 12:12 light cycle.

This study was carried out in strict accordance with the recommendations in the Guide for the Care and Use of Laboratory Animals, NRC 1996.  The protocol was approved by the Institutional Animal Care and Use Committee of Psychogenics, Inc. (PHS OLAW animal welfare assurance number A4471-01), an AAALAC International accredited institution (Unit #001213). 

***Apparatus***. Experiments were conducted using eight modified IntelliCage units (IC, New Behavior AG), each with a camera mounted on top of the cage for computer vision analysis. Intra-maze spatial cues were added to the environment by placing laminated striped paper on the outside of the cage walls, while three climbing structures (two rods, a cubic central object and a three step staircase) were placed inside the cage to provide an enriched topology.

IntelliCages have 4 corners that can be freely accessed through small openings, containing antenna that can detect the animals’ ID from the electronic chips. Inside each corner two recessed openings with computer-controlled doors and infrared sensors control access to two water bottles, and allow monitoring of nosepoking and approach to the bottles. Nosepokes are detected by the infra-red sensors whenever a mouse places its muzzle inside the nosepoke hole, such that the system can respond to a nosepoke by immediately opening a door directly behind the IR sensor, allowing the mouse to consume water from the bottle.

The cages were maintained either on a 12:12 light/dark cycle, with white light during the day and dim red light during the night, or with constant dim red light to allow video recording while simulating constant dark conditions from the perspective of the mice. The light intensity under red light was recorded at 7 lux using a photographic bandpass filter (LDP LLC, NJ) that eliminates long wavelength light frequencies not visible to mice [22]. Water was only available from within the corners, while food (14mg dustless precision pellets, Bio-Serv, NJ) and an alternative hydration source in the form of HydroGelTM (ClearH20, ME) packs was freely available on the cage floor at all times. Note that, even in the presence of hydrogel, mice generally appear motivated to obtain water.

## **Protocols**

***Habituation***: In this phase water is freely available in all four corners (Figure 1).

***Alternation***: During this phase subjects are required to alternate visits to two of the four corners (active corners) in order to gain access to water. Each mouse is assigned two adjacent active corners at random. Only one of the two nosepokes was activated in each corner, such that e.g. mice had to nosepoke on the left side in order to gain access to water in the right corner, or nosepoke to the right in the left corner (see Figure 1). After a correct alternation, a nosepoke in the correct recess resulted in the door opening and allowing access to the water bottle on that side. After 8 seconds, the door gently closed, preventing further access to water, unless the alternative active corner was visited. No penalty was imposed for initially nosepoking on the incorrect side. 

***Extinction*** *: * The protocol here is identical to the Habituation period, in which all corners provide free access to water, with both doors opening as a mouse enters any corner.

Both cohorts had been exposed to the apparatus once, 6 weeks or more prior to these 6 day studies. The animals in Cohort 1 were initially exposed at 36 weeks of age, 6 weeks prior to the current test at 42 weeks, while Cohort 2 were exposed at 24 weeks of age 36 weeks prior to the current test. Both groups of animals received 1 day of Habituation and 2 days of Alternation in these initial tests.

In the present studies, Cohort 1 was retrained for an initial 2 days on the Alternation protocol, then switching to Extinction for the remaining 4 days, with a 12:12 h light/dark cycle.   Cohort 2 remained on the Alternation protocol throughout the 6 days, with a reversal of the within-corner nosepoke rule occurring after 2 days, with red light throughout the study. Prior to entering the PhenoCube^TM^ system, all mice were water restricted in their home cages for 16 hours, to increase their motivation to interact with the IntelliCage corners.  

**Figure:**
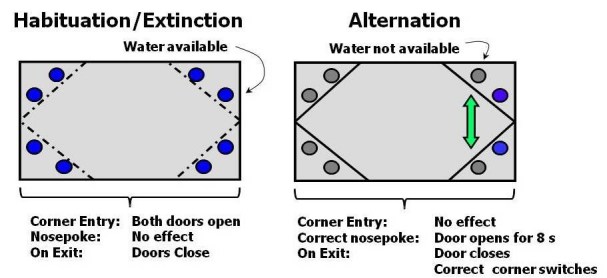
*Figure 1.* Cage plan and water access protocol during Habituation and Alternation phases. Cages have 8 available water bottles accessible through a hole that can be closed with a computer-operated gate. During the habituation phase all bottles are available. During the alternation phase mice need to enter the correct corners, which alternates between two locations, and nosepoke into the recess to open the gate and gain access to the bottle. 

***Cued Two-Choice Swim Tank Test*** *.* The cued two-choice swim test is a simple visual discrimination task that assesses learning and memory in rodents. Rodents are trained to swim toward the side of the tank signaled by a light positioned over the escape platform, the location of which varies from trial to trial relative to the animals’ starting position in the tank. Accordingly, any acquisition of this task clearly indicates that the animals are able to detect the cue light, such that they must have a functional visual system. Animals are placed in a rectangular tank (76 cm x 30.5 cm x 30.5 cm) filled with water maintained at 25°C ± 1°C, rendered opaque by the addition of non-toxic white paint. The escape platform (diameter of 7 cm) was located 0.5 cm below the surface of the water, on random sides, and its position is signaled by a light cue (a 25 Watt incandescent bulb). On each trial, mice were placed in the middle of the tank and allowed to swim until they found the platform or for a maximum of 1 min. Mice that did not reach the platform within 1 min were placed on it for 30 s. After each trial mice were placed in a pre-warmed holding cage placed on a warming pad. Mice received 8 trials per day, in 4 blocks of 2 trials, with training continuing for each mouse until it reached a criterion of 75% correct choices for 2 consecutive days. Correct choices were scored if the subject turned in the cued direction and climbed the platform, while swimming towards the other side was scored as incorrect.

***Data collection and analysis.*** Data were obtained from the detectors associated with the corners (namely, number of corner entries, location of corner entries, time present within a corner, number of nosepokes, and number of licks) on an individual basis. Computer vision data (namely locomotion, immobility, and rearing/climbing) was analyzed instead with cages of mice as the basic unit, such that groups of mice contributed a single datapoint. An alternation between the two active corners was included in the analysis only if it occurred less than 113-s of leaving an active corner. In analysis of behavioral measures related to the IntelliCage corner tasks (repeats, alternations, correct nosepokes, and exploratory visits), only data from mice which continued to make licking responses were included, since only these animals were presumably engaged in the learning task. In addition to corner visits and nosepokes, activity (locomotion, immobility, huddling, climbing and rearing) outside the corners was collected through a proprietary automated computer vision video scoring system (PsychoGenics Inc.). All data in these analyses (except for the corner learning task) was taken only from the final 5 complete 24 hour periods in the cage, starting from the entrained lights-on time on the second day. This rule was adopted to avoid any potentially confounding effects of cage novelty or thirst on the animals’ endogenous behavioral rhythms. 

Overall rate data from the 42 week old mice, tested under a fixed 12:12 light cycle, was analyzed via mixed factor ANOVA, using phase of the cycle (light vs. dark), genotype (WT vs. BAC TG) and testing day (1 to 5) as factors. Since the 60 week mice were tested under constant light conditions, with the circadian period of the mice being a dependant measure in the study, neither cycle phase nor testing day could be utilized as factors in these analyses, which were instead conducted by Student’s T-test on overall session data from the two groups. 

Analysis of the corner learning task, in which mice learned to nosepoke at one of two possible recesses within correct corners, was conducted in 12h bins from the scheduled start of the test sessions, irrespective of time and light cycle. In this analysis we compared the learning rate of the two genotypes.

For evaluation of circadian period, behavior was analyzed using ClockLab Analysis software (Actimetrics, Evanston, IL), and activity was quantifiedas the number of **behavioral counts** (defined appropriately for each measure analyzed) occurring during 10-min bins. The free-running period of behavioral rhythms and the rhythm amplitude was computed by using the chi-square periodogram. The proportions of activity during subjective day and night, as well as the total amount of activity per day were determined using the "activity profile" function for each mouse, plotted using the period of the animal; note that, in some cases, no period could be calculated for a given animal, with such animals dropped from the relevant analyses

In data from the two-choice swim tank task, the proportion of mice to reach the acquisition criterion (75% correct choices for 2 consecutive days) was assessed using Kaplan-Meier event analysis over the entire acquisition test period.

## **Results**

### ***PhenoCube*** ^***TM***^ ***system***

### ***Activity (Total Visits): Significant hypoactivity and lengthened circadian cycle in BAC TG mice.***

**Figure:**
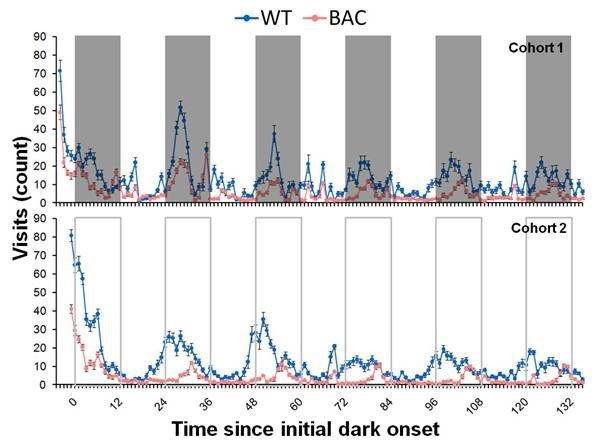
*Figure 2.  *Significant hypoactivity and lengthened circadian cycle in BAC TG mice is seen in the frequency of visits to the corners, presented here for BAC and WT mice as a function of session time (mean ± SE) for cohort 1 (normal light/dark cycle, top panel) and cohort 2 (constant dim red light, bottom panel). The 0 h mark was assigned to the time when the first dark cycle started (cohort 1) or should have started (cohort 2). 

BAC mice made fewer entries to the corners at both 42 and 60 weeks of age (Figure 2). In data from cohort 1, the phenotype was evident throughout the light-dark cycle, appearing more pronounced when the WT mice are more active in the dark periods. In analysis of cohort 1, broken down into light and dark periods, this effect reached significance via a genotype x light/dark phase interaction, while the analysis also revealed significant overall main effects of both genotype and light/dark phase, smallest *F*(1,51)=46.0, * p* < 0.0001. While there was also a significant main effect of testing day, along with a significant interaction between light/dark phase and test day, smaller *F*(4,204) = 60.5, *p*<0.0001, indicating reduced activity as the animals acclimated to the PhenoCube^TM^ system environment, these effects did not interact with genotype, larger * F*(4,204) = 2.20, all *ps* > 0.07.

Further examination of the data from cohort 1 suggested that the peak of activity of the BAC mice was progressively delayed as days in the apparatus passed (see Figure 2, upper panel), although chi-square periodogram, revealed no significant differences between the two groups in either period or period amplitude, larger *t*(49) = 0.78, all *ps >*0.4.

**Figure:**
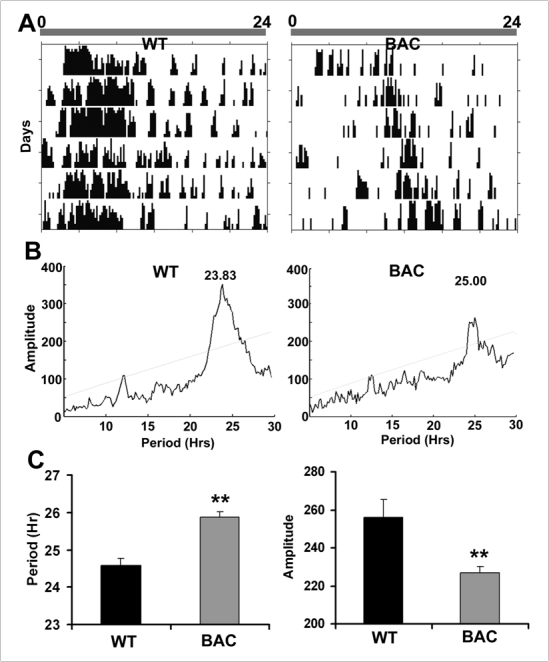
*Figure 3. *Periodogram and power analysis of visit frequency data from cohort 2 (from Figure 2, lower panel). **(A)** The bar above the actograms shows the continuous red light regimen for a 24h cycle. Actograms depict daily activity of representative WT and BAC mice. **(B)** Spectral analyses of Chi-square periodogram indicating the period and amplitude for each actogram in A. **(C) **Bar graphs showing the average period and amplitude (±SEM) for WT and BAC mice under constant red light; **p<0.01.

To further explore the circadian rhythms of these BAC HD mice, cohort 2 were tested under constant red light conditions.  Data from this experiment again revealed a highly significant reduction in overall activity in the BAC TG mice, *t*(61) = 11.3, *p* <  0.001, while also indicating a more pronounced shift in the activity peaks, as seen in the lower panel of Figure 2. This shift in phase was confirmed by chi-square periodogram for the number of corner entries, revealing that BAC mice had both a longer period and smaller rhythm amplitude than did WT mice, smaller * t*(60)= 2.82, *ps* < 0.01, with an average difference in period of 1 h 18 min (see Figure 3). 

### ***Increased licking behavior in BAC TG mice in cohort 1 only.***

In analysis of cohort 1, significantly increased licking behavior was detected in the BAC TG mice, along with significant effects of test day and of light/dark phase, each of which interacted both with genotype and with one another, smallest *F*(1,49) = 26.3, smallest *F*(4,196) = 2.91, all *ps *< 0.03. There was no significant three way interaction, *F*(4,196) = 1.64. Inspection of these data (not shown) did not reveal any clear pattern as a function of training days. Testing of cohort 2 at 60 w, by contrast, did not reveal any genotypic differences in licking behavior, *t*(61) = 1.46, *p* > 0.1, possibly reflecting the different test protocol in use. Circadian analyses of these data were not practical, due to long periods spent with no licking behavior observed in a high proportion of the mice.

### ***Corner Visit Duration: Increased visit duration in BAC TG mice.***

At both ages BAC mice spent considerably more time inside the corners on each visit during both tests (not shown; cohort 1, genotype *F*(1,51)=67.5, *p* < .0001; cohort 2, *t*(61) = 8.22, *p* < 0.0001). In the 42 week test, there were significant changes in visit duration and in the impact of light/dark phase across the test period, smaller * F*(4,204) = 3.296, *p* < 0.02, but no interactions involving genotype, all *F*s < 1.

No circadian analyses could be performed on this measure or the subsequent percentage measures, since the values were obtained using overall performance rates as denominators. 

### ***Reduced repeat visiting in BAC TG mice.***

BAC mice reentered visited corners less than did WT controls. In cohort 1the first cohort, the genotypic differences were more pronounced during the dark phase, when WT seem to make more reentries, *F*(1,49) = 11.8, *p* < 0.002, although the overall effect did not reach significance, * F*<1. There were also significant overall changes across test days and with light/dark phase, along with a day x phase interaction, smaller *F*(4,196) = 3.51, *F*(1,49) = 53.9, both *p*s < 0.01, but no three way interaction nor days x genotype interaction, larger *F*(4,196) = 1.29, both *ps* > 0.25. This effect was highly significant in overall analysis of cohort 2, *t*(35) = 4.78, *p* < 0.001. 

### ***Similar alternation performance in both genotypes.***

In entrained light/dark testing at 42 weeks, there was a trend towards decreased alternation performance during the light periods in the BAC TG mice, *F*(1,45)^1^ = 3.56, *p* < 0.07, along with a significant overall effect of light/dark phase, *F*(1,45) = 41.1, and a marginal interaction between training day and light/dark phase, *F*(4,180) = 2.30, * p* < 0.07. There were no other significant effects or interactions, * F*s < 1. At 60 weeks of age, tested under constant conditions, no genotype differences were apparent, *t*(35) = 0.998, *p* > 0.3, confirming that these were not reliable differences. 

***Reduced exploratory behavior in BAC TG mice*** *.*

BAC mice in cohort 1 made a lower proportion of visits to unreinforced corner locations (‘exploratory corners’) than did WT controls at the start of the session. This effect diminished with elapsing time in the cages, as indicated by a significant interaction between genotype and test day, * F*(4, 196) = 6.49, *p* < .0001, although the overall main effect did not approach significance, *F*<1. There was also a significant overall effect of days and a significant interaction between light/dark phase and days, smaller *F*(4,196) = 6.02, *p* < 0.0001, but no other effects or interactions approached significance, larger *F*(1,49) = 1.61, p > 0.2, *F*(4,196) = 1.60, p > 0.15. At 60 weeks, with testing under constant red light, the BAC TG mice again made a lower proportion of visits overall to the exploratory corners,* t*(35) = 3.08, *p* < 0.01.

### ***No deficits in nosepoke rule learning.***

Cohort 1 was trained over 36 h to nosepoke on only one of the two corner recesses to obtain reward. Both groups learned the correct side successfully, reaching around 80% correct responses, as indicated by a significant main effect of training bin, *F*(3,159)^1^= 31.4, *p* < 0.0001. There were no significant effects of genotype, though there was a trend towards improved overall performance in the BAC TG mice, *F*(1,53) = 3.70, *p* = 0.06, and a marginal interaction between genotype and training bin, *F*(3,159) = 2.12, *p* < 0.1. 

Cohort 2 had 48 h of training, plus 84 h of reversal (if the right nosepoke was correct, they had to switch to the left and vice versa). Again both groups reached about 80% correct first responses in both learning and reversal, with highly significant overall effects of training bin in both cases, * F*(3,96)^1^ = 13.4, *F*(6,192)^1^ = 18.5, * p* < 0.0001. In initial training, where the animals were thirsty, the BAC TG mice performed significantly better overall, *F*(1,32) = 8.55, *p* < 0.01, though this effect did not interact with training bin, *F*<1. There were no genotype differences in reversal performance, *F*(1,32) = 2.24, *F*(6, 192) = 1.30, p > 0.14, with this training occurring while the animals were not water restricted.

### ***Reduced locomotion in BAC TG mice.***

**Figure:**
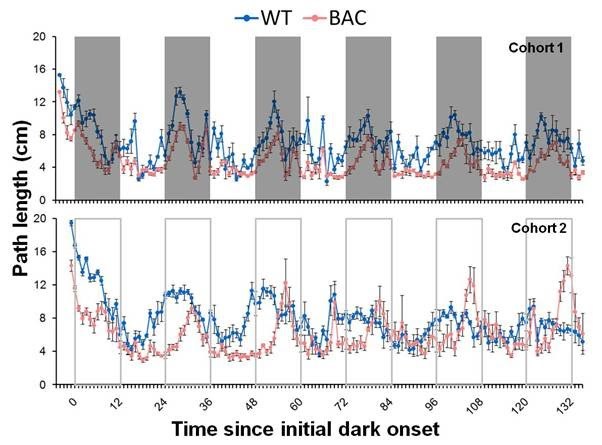
*Figure 4.*  Clear hypoactivity is also seen in BAC TG mice in the distance travelled within the cage, presented here as path length as a function of session time (mean ± SE of cage average) for cohort 1 (normal light/dark cycle, top panel) and cohort 2 (constant dim red light, bottom panel). The 0 h mark was assigned to the time when the first dark cycle started (cohort 1) or should have started (cohort 2). 

As illustrated in the upper panel of Figure 4, BAC mice covered less distance in the cage (outside the corners) than did WT controls at 42 weeks of age, *F*(1,6) = 73.6, *p* < 0.001, along with a significant overall decline in activity levels across the five days analyzed and a significant reduction in the overall effect of light/dark phase with day, smaller *F*(4,24) = 10.6, *p* < 0.0001. There was no interaction between day and genotype nor a significant three-way interaction between day, phase and genotype, larger *F*(4,24) = 1.93, both *ps* > 0.1. In comparison of the light and dark phases, significantly elevated overall activity in the dark period was detected, an effect with interacted with genotype, smaller *F*(1,6) = 23.4, *p* < 0.01. Although separate analysis of the two genotypes revealed a significant effect of phase in both groups, this interaction indicated that the difference was less pronounced in the BAC mice. Circadian evaluation of these data did not reveal any significant difference in period between the two genotypes, *t*(6) = 1.30, *p* > 0.2. 

Cohort 2 (Figure 4, lower panel) showed the same overall difference, *t*(6)= 4.56, *p* < 0.01, although an interesting pattern developed over days: whereas during the first three days BAC mice were generally hypoactive with respect to the WT mice, as WT mice habituated to the cage and showed less activity, the BAC mice reduced activity became less pronounced, with some peaks of locomotor activity showing the reversed pattern, that is, peaks of activity of BAC mice were more intense than those of WT controls during the last few days of testing. Note that the peaks of activity of the BAC groups were progressively shifted toward later times as expected from the circadian FF analysis (Figure 4), but in these data the period differences failed to reach significance, *t*(6) = 1.67, *p* > 0.1. This pattern of overall decreased distance covered and intense activity peaks at the oldest age tested was not the opposite image of immobility.

### ***Increased immobility in BAC TG mice.***

BAC TG mice consistently spent more time immobile at all time, at both ages tested. Analysis of these data for the light/dark cohort again revealed a significant main effect of genotype, *F*(1,6) = 16.5, *p* < 0.01, and a significant change in behavior across testing days, *F*(4,24) = 4.77, *p* < 0.01. While there was a significant interaction between genotype and light/dark phase, *F*(1,6) = 20.6, *p* < 0.005, and between test day and light/dark phase, *F*(4,24) = 2.87, *p* < 0.05, there was only a trend here towards overall differences between the light and dark periods, *F*(1,6) = 4.00, * p* < 0.1. There was no hint of a three way interaction, *F*(4,24) = 1.35, *p* > 0.25. Follow-up analyses of the two genotypes separately revealed a significant effect of light/dark phase only in the BAC transgenic animals, an impression consistent with circadian analysis which failed to reveal any sign of periodicity in these data for the WT mice.

Analysis of data from testing at 60 weeks of age, conducted under constant red light, also revealed a significant elevation of time spent immobile in the BAC mice, * t*(6) = 10.2, *p* < 0.001. Circadian analysis again failed to reveal any period in the WT mice, so no genotype comparison was possible.

### ***Significantly reduced rearing and climbing activity in BAC TG mice.***

All vertical activity was drastically reduced in the BAC mice (data not shown). Analysis of the 42 week test revealed a highly significant main effect of genotype, along with both a significant overall increase in activity in the dark periods and an interaction between light/dark phase and genotype, smallest * F*(1,6) = 59.4, *p* < 0.0005. There was also a significant reduction in overall activity across the 5 test days, along with an interaction between test day and light/dark phase, smaller *F*(4,24) = 9.05, *p* < 0.0005, but no interaction between test day and group, *F*(4,24) = 2.22, *p* = 0.098, nor a three-way interaction, * F*<1. Follow up analyses of the two genotypes again revealed significant effects of light/dark phase in both groups of mice. Circadian analysis of these data indicated that both genotypes had comparable periods under these conditions, *t*(6) = 2.05, *p* > 0.08.

Simpler analysis of the data from cohort 2, tested at 60w, again revealed a highly significant reduction in activity in the BAC mice, *t*(6) = 15.3, *p* < 0.0001, while circadian analysis of these data indicated significantly lengthened period in the BAC TG mice, *t*(6) = 7.62, *p* < 0.001, with the mean period being ~60 minutes longer in the mutant animals (not shown).

### ***No deficits in visual discrimination task (two-choice swim tank).***

**Figure:**
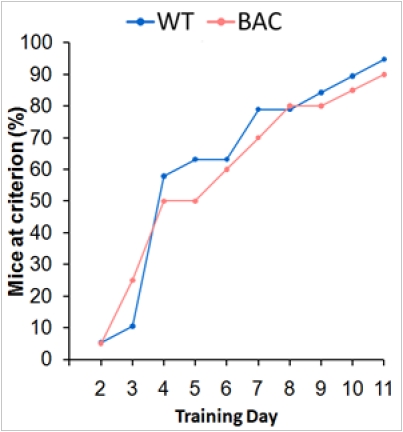
*Figure 5.*  No deficits are seen in acquisition of the cued swim tank, presented here as the percentage of BAC TG and WT mice reaching the learning criterion as a function of session number. 

Both BAC and WT mice successfully learned to swim toward a light cue as shown by their performance on this task, illustrated in Figure 5 as the proportion of mice which acquired the visual discrimination as a function of training session (Kaplan-Meier, *p *> 0.8). These data clearly indicate that these BAC TG animals are able to detect light in their environment, such that any deficit in circadian entrainment is not purely visual in origin.  

## **Discussion**

We have shown here that, in the group housed condition used in this study, BAC HD mice have a significant lengthening of the circadian cycle, accompanied by substantial changes in motor activity. In addition to these primary results, we also present data illustrating the comprehensive phenotype obtained from our fully automated screening system. Given that our system involved testing groups of mice, the possibility exists that the period measured here may have been modified by the groups’ behavior. Accordingly, our findings need to be expanded with the analysis of individual mice in a classical circadian setting.

 The period lengthening observed in the BAC mice differs from that observed in the R6/2 mice, which initially showed a shortening of the period, followed by a more pronounced deficit, namely, a disintegration of the circadian cycle [3]. These differences may be due to a different pattern of neuronal dysfunction or gene expression dysfunction; for instance R6/2 mice show disruption in mPer2 and mBmal1 expression in the SCN. Under constant light conditions, mPer2 mutant mice show a short circadian period initially, followed by gradual loss of rhythmicity [23], resembling the pattern seen in the R6/2 mice, whereas Bmal1 knockout mutants are immediately arrhythmic in DD. Knocking out either clock or Per1 genes result in shortening of the circadian cycle [24] [25]. Other changes and mutations in genes involved in regulatory aspects of the circadian rhythm may lead to lengthening of the period (e.g., Hsf1 or CKIdelta knockout [26] [27], Per3 polymorphism [28]). Therefore shortening or lengthening of the circadian machinery, in R6/2 and BAC, respectively, may be a reflection of a different pattern of progressive gene expression changes secondary to the loss of normal huntingtin function. This type of dysfunction is also seen in patients with Huntington’s disease, who are known to have significantly abnormal patterns of day/night activity[3].

The SCN is the main brain clock pacemaker, serving to synchronize the phase of rhythms in the rest of the body, and to set their phase to local environmental cues, of which the light-dark cycle is the most important.  Photic signals that activate retinal ganglion cells project to the SCN via the retino-hypothalamic track. We explored the possibility that circadian deficits developed secondarily to a loss in photic responses.  Our visual discrimination study, however, suggests that this function is not impaired in the BAC mice. Moreover, BAC mice showed normal entrainment under a 12:12 h light-dark cycle, suggesting that the light was able to entrain their pacemakers.

Since the discovery of the mutation causing HD [29], many mouse models of HD have been generated. Idiosyncratic differences between models indicate they recapitulate different aspects of the human disease. Finding core dysfunctions that are common to various mouse models and humans enhances the chances that therapies based on such common pathological endpoints can be successful during translation from the bench to the clinic.

## **Acknowledgements**

BAC HD mice were provided courtesy of X. William Yang (UCLA David Geffen School of Medicine, Los Angeles CA). We are grateful to Margaret Robotham for statistical analysis.  

## **Funding information**

Supported by NIH grants NS37919 and MH075045 (to RS) and by the CHDI foundation.

## **Competing Interests**

Stephen Oakeshott, Igor Filippov, Carol Murphy, David Connor, Russell Port, Ahmad Paintdakhi, Liliana Menalled, Sylvie Ramboz and Dani Brunner are all employed by PsychoGenics, Inc., a for-profit institution. The authors have declared that no further competing interests exist.
